# Medicines and Meddlesomeness.—I

**Published:** 1922-10

**Authors:** R. W. Harris

**Affiliations:** (formerly Assistant Secretary, responsible for the administration of National Health Insurance Medical Benefits, Ministry of Health)


### MEDICINES AND MEDDLESOMENESS.—I.

**By R. W. HARRIS (formerly Assistant Secretary, responsible for the administration of National Health Insurance Medical Benefits, Ministry of Health).**

A MEDDLER is, I understand, one who interferes or busies himself officiously in the affairs of others. It will also be found, on examination, that he has an infinite capacity for muddling things. I suppose that more nonsense has been talked or written by meddlers from time to time on the subject of medicines for insured persons than on most questions relating to National Health Insurance ; it is straining politeness in many instances to attribute some of the misstatements to muddle, and not to use a harsher term.

Writers in papers which claim almost a monopoly of light and intelligence ; writers in other papers wherein they say if you see a statement it is so ; others who pose as guide, philosopher and friend to the simple-minded general practitioner—these, and yet others who deliver themselves in the more spacious atmosphere of the public platform, have repeated, at different times, statements about the supply of medicines to insured persons which are simply untrue. I remember once a noisy critic of the administration of the National Health Insurance system who made a series of statements to an interviewer which could have been set out categorically and then have written against each item of "intelligence"—"This is untrue." To have done so would have been merely to give him an advertisement; it is also not improbable that he would have had the last word. Reiteration carries conviction, and facts spoil stunts.

Of medicines for the insured it has been said that if a doctor writes any prescription for more than a given amount, he will be fined. The favourite limit of cost for the "stunt" writer is a shilling; "The Nimble Shilling" was the heading to one article. Others insist that a doctor is simply not allowed to think of what is good for the patient, but that he must apply his mind solely to the question of the cost of the ingredient. It is conceded, probably, that he is expected to give the patient something not altogether irrelevant to the complaint; but the dominating consideration is to be Cost, not Cure. Another critic, this time a prominent speaker in an important deputation to the Minister, asserted that it was "common knowledge" that panel doctors, in treating their patients, were allowed to choose one of twelve mixtures only. This critic, I believe, really belonged to the genus Muddler; he was not dishonest, except in so far as it is dishonest to avoid inquiry before making statements which carry with them a certain dramatic effect.

The explanation of the "twelve mixtures" observation could only be that in an earlier period there was an arrangement, since abandoned, under which the rates of payment to chemists for their services in dispensing medicines were lower in the case of certain mixtures (limited to twelve) which were agreed locally, by doctors and chemists, to be capable of being kept in stock for a reasonable period without deterioration. But the doctor, so far from being limited to the prescribing of these mixtures, had the whole range of the British Pharmacopœia, and the British Pharmaceutical Codex, from which to select medicines appropriate to the condition of his patient. The other class of misstatement is more difficult to place; it is so remote from anything which really occurs under the Insurance scheme. It can only be traced to the fact that the doctor's free range over the field of drugs is limited by one or two important considerations. Thus, he is expected, in the case of two drugs which, in the treatment of a given complaint, are of equal therapeutic value, to select that one which is less expensive. It is required of him that when one bottle of medicine has been taken, or half-a-dozen pills consumed, by a patient, he shall consider whether a second bottle is needed, or, whether a further box of pills should be handed out to the patient, as the case may be. Not in so many words, of course; the regulations state that if it appears that the cost of the medicines supplied to a doctor's patients is "in excess of what may reasonably be necessary for their adequate treatment," he may be invited to furnish an explanation, and in appropriate cases pecuniary penalties for extravagance may follow. "If it appears"—to whom? Not to any body of laymen, not to the Insurance Committee, though it contains representative medical men, and not to the officials of the Ministry. It is the doctor's own fellow-practitioners in the county or the county borough, elected to the Panel Committee by the panel doctors alone, who are entrusted with this check, which is imposed in the interest of those who provide the funds for Medical Benefit—the insured persons themselves, their employers and the over-burdened taxpayer. The Panel Committee cannot bring the question of reasonableness of the cost of a doctor's prescribing to a better practical test than the average cost of all the doctors in the county or county borough. Even then, if the individual doctor's *average* exceeds the general *average*, the case is one merely, in the first instance, for inquiry.

The meddlers, to whom I have referred, overlook, or suppress, the word "average," and manage to convey a perfectly grotesque idea of what is in fact being done. I hope to be allowed, in another article, to deal more fully with the question of the working of the machinery for checking excessive prescribing, but, in the meantime, I would ask those who criticise the system two questions: (1) Are 15,000 doctors who write prescriptions for 15,000,000 insured persons to be allowed a free hand—*i.e.*, to make uncontrolled demands on insurance and public funds? (2) If the answer is that there must be some check, can a better system, with equal economy, be devised than that of entrusting the check to the doctors themselves?

I address these questions to those who say, or imply, that the unfortunate insured person is being deprived of good, effective medicine because those in authority have decided that cheapness is all that matters. The "meddlesomeness" of these people is aggravated by the fact that their utterances are to be found in the pages of those very journals whose voice on Economy in public expenditure, when they are dealing with it *in the mass*, is loud indeed. How far they are doing a service or a disservice to the workers of this country in encouraging them to clamour for more medicine I will not venture to say, lest I should be placed in the class of meddlers. It is a question for medical men themselves to answer, and I observe from another article in this issue that one at least of them is disturbed because the National Health Insurance system is not weaning the worker from the 8 oz. bottle. I content myself with observing that the bottle of medicine is still a fearful and wonderful mystery. I have read that the word "medicine" is used in translating certain terms in the languages of the American aborigines, which denote not only "medicine" proper, but anything the operation of which they do not comprehend—that is, anything mysterious, supernatural, sacred. The first attempts at medical practice seem to have been made by, or under the direction of, priests. Is it that the critics writing in papers which loudly call for economy in other directions, feel that it is courting disaster to interfere in mysteries, or that the sum of £1,500,000 which is being spent on drugs for insured persons is, after all, an unconsidered trifle?

